# Predictive factors of the pharmacological action of tolvaptan in patients with liver cirrhosis: a post hoc analysis

**DOI:** 10.1007/s00535-016-1233-x

**Published:** 2016-07-05

**Authors:** Isao Sakaida, Shuji Terai, Koji Nakajima, Yoshiyuki Shibasaki, Sayaka Tachikawa, Hidetsugu Tsubouchi

**Affiliations:** 1 Yamaguchi University Graduate School of Medicine, Ube, Yamaguchi Japan; 2 Division of Gastroenterology and Hepatology, Graduate School of Medical and Dental Sciences, Niigata University, Niigata, Japan; 3 Department of Medical Affairs, Otsuka Pharmaceutical Co., Ltd., 2-16-4 Konan, Minato-ku, Tokyo, 108-8242 Japan

**Keywords:** Tolvaptan, Liver cirrhosis, Initial urine volume, Ascites-related clinical symptom, Predictive factor

## Abstract

**Background:**

Tolvaptan has been approved in Japan for the treatment of hepatic edema. An important consideration in providing a clinical benefit to patients with liver cirrhosis is the improvement of ascites-related clinical symptoms. In the present post hoc analysis, we aimed to identify factors that were predictive of the potency of tolvaptan, and to examine the relationship between changes in initial urine volume and improvement in ascites-related clinical symptoms.

**Methods:**

This post hoc analysis was based on three previous phase 2 and 3 clinical trials of tolvaptan in patients with liver cirrhosis. Predictive factors associated with a change in initial urine volume were identified. A change of ≥500 mL from baseline confirmed the pharmacological action of tolvaptan treatment. The relationship between the change in initial urine volume and improvement in ascites-related clinical symptoms was also examined.

**Results:**

A total of 152 patients were enrolled in this study. Body weight and BUN were identified as predictive parameters. Among patients with a change in initial urine volume of ≥500 mL, 75 % demonstrated improvement in ascites-related clinical symptoms, while no improvement was seen in those with a change of <500 mL. None of the patients with initial urine volume of <500 mL showed resolution of symptoms.

**Conclusions:**

Change in urine volume was affected by both baseline body weight and BUN in tolvaptan-treated subjects. Higher urine output was associated with improvements in ascites-related clinical symptoms.

## Introduction

The development of ascites is a major complication in patients with liver cirrhosis, associated with reduced quality of life and decreased survival [1]. The European Association for the Study of the Liver (EASL) [2] and the American Association for the Study of Liver Diseases (AASLD) [3] have provided practice guidelines for the management of ascites. Most patients are properly managed with diuretic treatment as a therapeutic option for controlling ascites. Among these agents, furosemide is useful for improving hypervolemic status, but is commonly associated with electrolyte imbalance, progressive renal failure, and worsening hepatic encephalopathy [4–8]. Evidence regarding both the advantages and disadvantages of conventional diuretics have been described in detail in the guidelines. However, with regard to drug development, over the past several decades, not a single effective compound has been launched as a diuretic.

Tolvaptan, an arginine vasopressin V_2_ receptor antagonist, has been approved for the treatment of patients with hyponatremia, hyponatremia secondary to syndrome of inappropriate antidiuretic hormone, heart failure related to volume overload, and autosomal dominant polycystic kidney disease. Tolvaptan was also approved in 2013 in Japan for the treatment of hepatic edema. The drug acts as an aquaretic agent, without the risk of conventional diuretic-induced complications [9]. In phase 2 and 3 clinical trials, tolvaptan was found to significantly reduce body weight and ascites volume, increase urine volume, and improve ascites-related clinical symptoms in liver cirrhosis patients with volume overload who had insufficient response to conventional diuretics [10–12]. However, not all patients have been found to benefit from tolvaptan; thus the identification of factors affecting the potency of the drug among patients is important. Recent studies have investigated predictive factors of the response and the occurrence of hypernatremia in tolvaptan-treated heart failure patients with volume overload [10, 11]. Unfortunately, data on tolvaptan in patients with liver cirrhosis is limited, given the brief period since approval. Therefore, an exploration of predictive factors with regard to the pharmacological action of tolvaptan is a meaningful and urgent need for liver cirrhosis patients with ascites.

In addition, liver cirrhosis is often accompanied by malnutrition [12], and the deteriorating conditions associated with ascites lead to reduced activity and motivation in patients. As the ultimate therapeutic option, liver transplantation is recommended; however, most patients have no indication, and have difficulty finding a donor. In these cases, the therapeutic management of ascites to improve symptoms is an important clinical goal for improving quality of life. Although change in body weight is a surrogate marker for evaluating diuretics, the diuretic effect is mainly a pharmacological action, and the relationship between diuretic effect and change in symptoms is not clear. Therefore, examining the effectiveness of tolvaptan for improving ascites-related clinical symptoms may provide important information of clinical benefit to patients with liver cirrhosis.

In the present post hoc analysis, through the integration of data from previous clinical trials, we aimed to identify factors that were predictive of the pharmacological action of tolvaptan in patients, based on change in initial urine volume. In addition, we examined the relationship between changes in initial urine volume and improvement in ascites-related clinical symptoms.

## Methods

### Patients

This post hoc analysis was based on three clinical trials of tolvaptan in cirrhotic patients with ascites [10–12], in which urine volumes and symptoms were analyzed as evaluation parameters. The trials were conducted in Japan to obtain an indication for tolvaptan for the treatment of hepatic edema. Data from the trials that were analyzed in this post hoc analysis included the pharmacokinetic and pharmacodynamic profiles (PK/PD) [10], the safety and efficacy of ≥7 consecutive days of treatment after dose escalation (escalating dose) [11], and the efficacy and safety of treatment for 7 consecutive days (pivotal) [12] . Eligible patients had persistent ascites despite combination therapy with conventional diuretics (a loop diuretic and an anti-aldosterone agent) prior to the start of the trials.

These clinical trials were conducted in accordance with the ethical principles set forth in the Declaration of Helsinki [13] and in compliance with good clinical practice guidelines [14]. The protocols were approved by the institutional review board at each trial site. All patients provided written informed consent. The trials are registered on ClinicalTrials.gov (NCT01114828 [10], NCT01048788 [11], and NCT01050530 [12]).

### Objectives

The objectives of the present post hoc analysis in patients with ascites who received the trial drug were to identify baseline parameters that could be used to predict the pharmacological action of tolvaptan, to evaluate changes in ascites-related clinical symptoms from baseline to day 7 based on changes in initial urine volume of ≥500 or <500 mL, and to evaluate changes in body weight and serum sodium level from baseline to day 1 based on urine volume stratification.

According to EASL guidelines, the maximum recommended rate of weight reduction during diuretic therapy is 0.5 kg/day in patients without edema [2]. Change in body weight has been used as a surrogate marker for evaluating the efficacy of diuretics. However, short-term outcomes of diuretic treatment involve increased urine volume and reduced body weight. Thus, we regarded a change in initial urine volume of ≥500 mL from baseline as an alternative marker to confirm the pharmacological action of tolvaptan.

### Study design

Inclusion and exclusion criteria were similar among the three trials. The present post hoc analysis integrated patients with liver cirrhosis from all three trials who had received oral administration of 7.5 mg tolvaptan once daily. Details of the clinical trial designs were published previously [10–12]. Tolvaptan at 7.5 mg once daily is the approved dose for the treatment of hepatic edema in Japan.

Baseline was defined as a 3-day observation period before administration of tolvaptan. Initial urine volume was defined as the urine volume collected during the first 24 h after initiation of treatment with tolvaptan. Baseline urine volume was defined as the 24-h cumulative urine volume collected during the 3-day observation period.

### Evaluations

Baseline patient characteristics and demographics (predictive factors) included sex, age, Child-Pugh classification [15], presence of hepatocellular carcinoma, dose of diuretics (as furosemide <40 or ≥40 mg), body weight, abdominal circumference, creatinine (Cr), blood urease nitrogen (BUN), serum albumin (Alb), total bilirubin (T-Bil), alanine aminotransferase (ALT), aspartate aminotransferase (AST), serum sodium (Na), serum potassium (K), serum osmolality, systolic blood pressure, and 24-h accumulating urine volume. Pharmacological action of tolvaptan was defined as a change in urine volume from baseline to day 1 of ≥500 mL.

Ascites-related clinical symptoms included bloating, loss of appetite, malaise, sensation of pressure when in a decubitus position, difficulty breathing, and general state, and were assessed at baseline and again on day 7. Clinicians questioned patients regarding the presence of ascites-related clinical symptoms at baseline and any change in symptoms by day 7, which patients assessed as “resolved”, “improved”, “unchanged” or “worsened”. Resolution or improvement of symptoms on day 7 was determined according to changes in initial urine volume of ≥500 or <500 mL. "General state" was the term used to evaluate any change in the patient’s impression of the study drugs.

Changes in body weight and serum sodium level from baseline to day 1 were also evaluated according to the above-mentioned cutoff values. In addition, these parameter levels were assessed according to the identified predictive factor.

### Statistical analysis

The full data set included all enrolled patients who received at least one dose of tolvaptan. Patients with missing baseline data or initial urine volume were omitted. Continuous data were analyzed using Student’s *t* test and are expressed as mean ± standard deviation (SD). Categorical data were analyzed using Fischer’s exact test and are expressed as number (%). Baseline parameters predictive of the greatest change in initial urine volume were investigated by univariate analysis. Significant parameters were further analyzed using a stepwise multiple logistic regression model. A value of ≥500 mL was determined as the cutoff point for defining a change in initial urine volume. Receiver operating characteristic (ROC) curve analysis was used to calculate the cutoff point and area under the curve (AUC) to the continuous data [16]. Data were expressed as odds ratio (OR), 95 % confidence interval (CI), and *P* value.

A two-tailed *P* value of <0.05 was considered statistically significant. All statistical analyses were performed using JMP^®^ 11 and SAS version 9.4 software (SAS Institute Inc., Cary, NC, USA).

## Results

### Patients

**Fig. 1:**
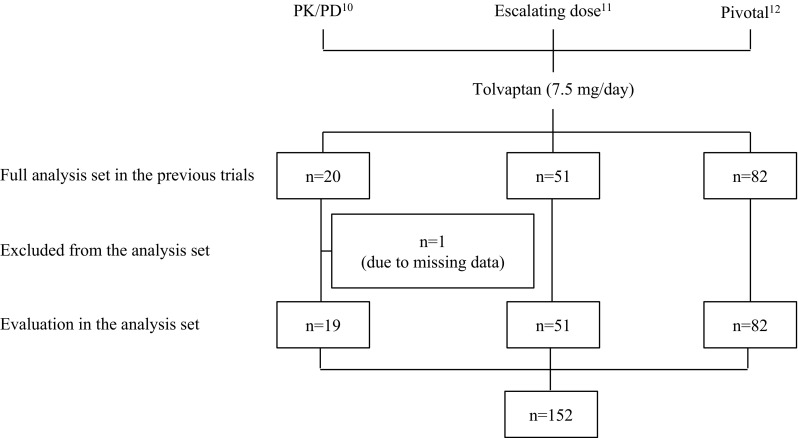
Enrolled patients and analysis set. Data are expressed as number of patients. *Superscript numbers* represent reference citations. *PK/PD* pharmacokinetics/pharmacodynamics

A total of 153 patients were enrolled in this post hoc analysis (Fig. 1); 152 were analyzed for efficacy, as one patient was excluded due to missing baseline urine volume data. Baseline characteristics and demographic data are shown in Table 1. Of the 152 patients, 114 (75 %) responded to tolvaptan treatment with a change in initial urine volume ≥500 mL, whereas 38 patients (25 %) experienced a change in urine volume <500 mL.

**Table 1:** Baseline characteristics and demographic data for patients with liver cirrhosis included in the present post hoc analysis (*n* = 152)

Feature	Value
Age [mean ± SD (years)]	65 ± 9
Sex, male [*n* (%)]	101 (66.4)
Child-Pugh, B [*n* (%)]	81 (53.3)
HCC, yes [*n* (%)]	42 (27.6)
Concomitant use of loop diuretics ≥40 mg [*n* (%)]	139 (91.4)
Body weight [mean ± SD (kg)]	60.2 ± 11.8
Abdominal circumference [mean ± SD (cm)]	90.4 ± 10.3
Serum creatinine [mean ± SD (mg/dL)]	1.02 ± 0.37
BUN [mean ± SD (mg/dL)]	23.8 ± 11.3
Serum albumin [mean ± SD (g/dL)]	2.8 ± 0.5
Total bilirubin [mean ± SD (mg/dL)]	1.6 ± 0.9
ALT [mean ± SD (IU/L)]	49.5 ± 28.2
AST [mean ± SD (IU/L)]	27.7 ± 17.3
Serum sodium [mean ± SD (mEq/L)]	134.8 ± 4.5
Serum potassium [mean ± SD (mEq/L)	4.1 ± 0.6
Serum osmolality (mOsm/L)]	283.3 ± 10.3
Blood pressure [mean ± SD (mmHg)]	111.9 ± 15.1
Urine volume [mean ± SD (mL)]	1464 ± 700

Concomitant use of loop diuretics is converted to furosemide substantial amount

*HCC* hepatocellular carcinoma, *BUN* blood urea nitrogen, *ALT* alanine aminotransferase, *AST* aspartate aminotransferase, *n* number of patients, *SD* standard deviation

### Baseline parameters predictive of the pharmacological effects of tolvaptan

**Fig. 2:**
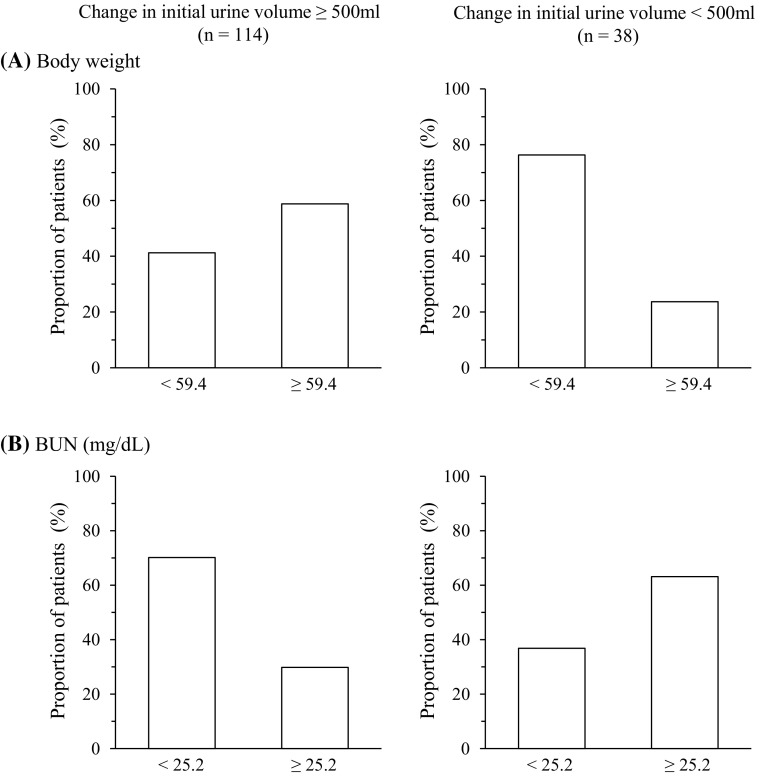
Proportions of patients with increase in initial urine volume of ≥500 or <500 mL from baseline according to stratification of **a** body weight of 59.4 kg and **b** blood urea nitrogen (BUN) of 25.2 mg/dL at baseline. Data are expressed as percentage

In univariate analysis, subjects with an initial change in urine volume >500 mL were more likely to be younger (64 vs. 65, *P* = 0.005) and heavier (62 vs. 56 kg, *P* = 0.007), with lower serum creatinine (0.97 vs. 1.18, *P* = 0.002), lower BUN (22 vs. 29, *P* = 0.001), higher total bilirubin (1.7 vs. 1.2, *P* = 0.015), and lower serum osmolality (282 vs. 287, *P* = 0.024; Table 2). In multivariate analysis, baseline body weight was a significant predictive factor (OR 1.05: 95 % CI 1.01–1.09; *P* = 0.0320; Table 3). In a stepwise multivariate regression analysis using all significant variables in the univariate analysis (Table 3), baseline body weight (OR 1.05: 95 % CI 1.01–1.09: *P* = 0.0143) and BUN (OR 0.95: 95 % CI 0.92–0.98; *P* = 0.0051) were significantly associated with change in initial urine volume. The cutoff points for body weight and BUN were 59.4 kg and 25.2 mg/dL, and AUC were 0.66 and 0.69, in graphical display of the ROC curves, respectively. Fifty-nine percent (67/114) of patients with body weight ≥59.4 kg experienced a change in initial urine volume ≥500 mL, and 24 % (9/38) with body weight ≥59.4 kg experienced a change of <500 mL (Fig. 2a), while 70 % (80/114) of patients with BUN <25.2 mg/dL experienced a change in initial urine volume of ≥500 mL, and 37 % (14/38) with a BUN <25.2 mg/dL experienced a change of <500 mL (Fig. 2b).

**Table 2:** Factors at baseline predictive of the pharmacological action of tolvaptan based on change in initial urine volume of ≥500 or <500 mL from baseline using univariate analysis

	≥500 mL (*n* = 114)	<500 mL (*n* = 38)	*P* value^†^
Age [mean ± SD (years)]	64 ± 9	69 ± 9	0.0052*
Sex, male [*n* (%)]	77 (67.5)	24 (63.2)	0.6926
Child-Pugh, B [*n* (%)]	56 (49.1)	25 (65.8)	0.0918
HCC, yes [*n* (%)]	33 (28.9)	9 (23.7)	0.6759
Concomitant use of loop diuretics ≥40 mg [*n* (%)]	105 (92.1)	34 (89.5)	0.7378
Body weight [mean ± SD (kg)]	61.6 ± 11.8	55.7 ± 10.9	0.0070*
Abdominal circumference [mean ± SD (cm)]	91.2 ± 10.2	88.0 ± 10.6	0.1015
Serum creatinine [mean ± SD (mg/dL)]	0.97 ± 0.34	1.18 ± 0.41	0.0021*
BUN [mean ± SD (mg/dL)]	22.1 ± 10.5	28.9 ± 12.4	0.0012*
Serum albumin [mean ± SD (g/dL)]	2.8 ± 0.5	2.9 ± 0.5	0.0936
Total bilirubin [mean ± SD (mg/dL)]	1.7 ± 0.9	1.2 ± 0.9	0.0145*
ALT [mean ± SD (IU/L)]	51.2 ± 29.7	44.5 ± 22.8	0.2066
AST [mean ± SD (IU/L)]	28.0 ± 17.4	26.9 ± 17.1	0.7402
Serum sodium [mean ± SD (mEq/L)]	134.6 ± 4.6	135.2 ± 4.1	0.5421
Serum potassium [mean ± SD (mEq/L)	4.0 ± 0.5	4.16 ± 0.68	0.2112
Serum osmolality (mOsm/L)]	282.2 ± 10.4	286.5 ± 9.4	0.0236*
Blood pressure [mean ± SD (mmHg)]	111.3 ± 15.0	113.7 ± 15.7	0.4032
Urine volume [mean ± SD (mL)]	1506 ± 671	1339 ± 779	0.2037

Data are expressed as number (%) or mean ± SD, stratified according to change in initial urine volume from baseline

Concomitant use of loop diuretics is converted to furosemide substantial amount

*HCC* hepatocellular carcinoma, *BUN* blood urea nitrogen, *ALT* alanine aminotransferase, *AST* aspartate aminotransferase

^†^Student’s *t* test for continuous data, and Fischer’s exact test for categorical data

* *P* < 0.05

**Table 3:** Evaluation of results using multiple regression analysis and stepwise method for the predictive factors

Predictive factor	*P* value	Odds ratio	95 % CI
Multiple logistic regression analysis			
Intercept	0.5246		
Age	0.1233	0.96	0.91–1.01
Body weight	0.0320	1.05	1.01–1.09
Serum creatinine	0.0790	0.24	0.05–1.19
BUN	0.8186	0.99	0.94–1.05
Total bilirubin	0.1737	1.44	0.87–2.50
Serum osmolality	0.7441	0.99	0.94–1.04
Stepwise			
Intercept	0.7641		
Body weight	0.0143	1.05	1.01–1.09
BUN	0.0051	0.95	0.92–0.98

Statistical inference obtained by multiple logistic regression analysis

*95* *% CI* 95 % confidence interval

### Changes in ascites-related clinical symptoms according to change in initial urine volume

The proportion of patients with resolved or improved ascites-related clinical symptoms by day 7 according to change in initial urine volume is reported in Table 4. Among patients showing an initial change in urine volume ≥500 mL, a higher proportion of patients reported improvement or resolution of symptoms including bloating [70.1 % (61/87) or 10.3 % (9/87), respectively], loss of appetite [48.7 % (19/39) or 7.7 % (3/39), respectively], malaise [57.4 % (35/61) or 4.9 % (3/61), respectively], sensation of pressure when in decubitus position [63.6 % (35/55) or 12.7 % (7/55), respectively], and difficulty breathing [69.6 % (16/23) or 4.3 % (1/23), respectively]. An improvement in their general state was reported by 80.2 % (85/106) of patients.

**Table 4:** Improvement rates of ascites-related clinical symptoms

Symptoms	≥500 mL				<500 mL			
	Total %	PK/PD %	Escalating dose %	Pivotal %	Total %	PK/PD %	Escalating dose %	Pivotal %
Improvement								
Bloating	70.1 (61/87)	58.3 (7/12)	76.7 (23/30)	68.9 (31/45)	36.8 (7/19)	0.0 (0/2)	50.0 (4/8)	33.3 (3/9)
Loss of appetite	48.7 (19/39)	66.7 (2/3)	50.0 (5/10)	46.2 (12/26)	18.2 (2/11)	0.0 (0/2)	0.0 (0/1)	25.0 (2/8)
Malaise	57.4 (35/61)	37.5 (3/8)	50.0 (9/18)	65.7 (23/35)	31.6 (6/19)	0.0 (0/2)	28.6 (2/7)	40.0 (4/10)
Sensation of pressure in decubitus position	63.6 (35/55)	50.0 (4/8)	57.9 (11/19)	71.4 (20/28)	40.0 (6/15)	0.0 (0/1)	33.3 (2/6)	50.0 (4/8)
Difficulty breathing	69.6 (16/23)	66.7 (2/3)	55.6 (5/9)	81.8 (9/11)	33.3 (2/6)	−(0/0)	50.0 (1/2)	25.0 (1/4)
General state	80.2 (85/106)	61.5 (8/13)	88.6 (31/35)	79.3 (46/58)	45.2 (14/31)	33.3 (1/3)	45.5 (5/11)	47.1 (8/17)
Resolution								
Bloating	10.3 (9/87)	8.3 (1/12)	3.3 (1/30)	15.6 (7/45)	0.0 (0/19)	0.0 (0/2)	0.0 (0/8)	0.0 (0/9)
Loss of appetite	7.7 (3/39)	0.0 (0/3)	0.0 (0/10)	11.5 (3/26)	0.0 (0/11)	0.0 (0/2)	0.0 (0/1)	0.0 (0/8)
Malaise	4.9 (3/61)	0.0 (0/8)	5.6 (1/18)	5.7 (2/35)	0.0 (0/19)	0.0 (0/2)	0.0 (0/7)	0.0 (0/10)
Sensation of pressure in decubitus position	12.7 (7/55)	0.0 (0/8)	10.5 (2/19)	17.9 (5/28)	0.0 (0/15)	0.0 (0/1)	0.0 (0/6)	0.0 (0/8)
Difficulty breathing	4.3 (1/23)	0.0 (0/3)	11.1 (1/9)	0.0 (0/11)	0.0 (0/6)	−(0/0)	0.0 (0/2)	0.0 (0/4)

*PK/PD* pharmacokinetics/pharmacodynamics

### Changes in body weight and serum sodium level according to change in initial urine volume

**Fig. 3:**
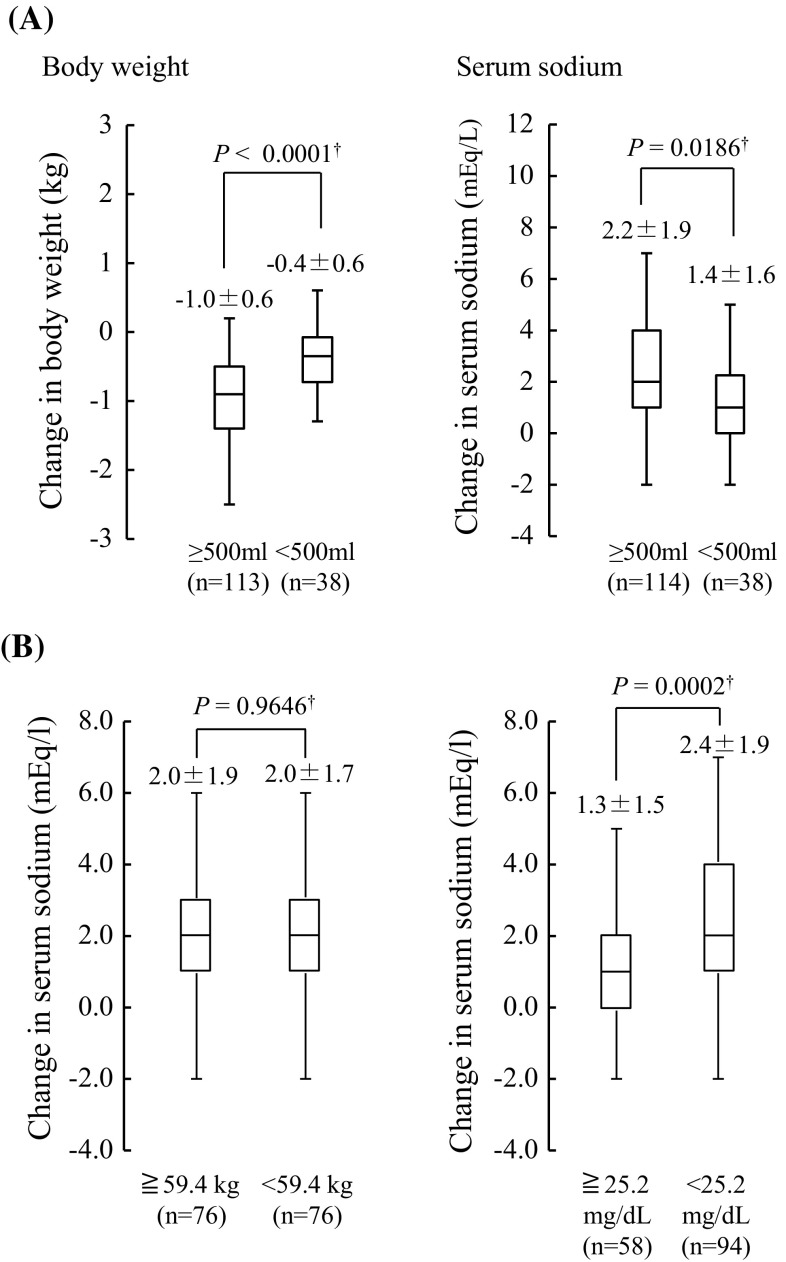
**a** Box plots showing changes in body weight and serum sodium level from baseline to day 1 based on change in initial urine volume of ≥500 or <500 mL. **b** Box plots showing changes in serum sodium level from baseline to day 1 based on body weight of 59.4 kg and blood urea nitrogen (BUN) of 25.2 mg/dL at baseline. Data are expressed as mean ± standard deviation. Statistical analysis was performed using the Student *t* test

The changes in body weight and serum sodium levels are summarized as box plots in Fig. 3a. Changes in body weight from baseline to day 1 in patients with a change in initial urine volume of <500 and ≥500 mL were −0.4 ± 0.6 kg and −1.0 ± 0.6 kg, respectively (*P* < 0.0001). The change in serum sodium level from baseline to day 1 was 1.4 ± 1.6 mEq/L in patients with a change in initial urine volume of <500 mL, and 2.2 ± 1.9 mEq/L in those with a change ≥500 mL (*P* = 0.0186). These differences were statistically significant.

To determine whether body weight and BUN were associated with improved pharmacological action of tolvaptan, we evaluated the change in serum sodium levels from baseline to day 1, stratified by baseline body weight of ≥59.4 and <59.4 kg and by baseline BUN of ≥25.2 and <25.2 mg/dL. Body weight was not associated with a change in serum sodium (2.0 ± 1.9 mEq/L for body weight ≥59.4 kg, vs. 2.0 ± 1.7 mEq/L for <59.4 kg; *P* = 0.9646), while low BUN values were associated with statistically significant improvements in serum sodium (1.3 ± 1.5 mEq/L for BUN ≥25.2 mg/dL vs. 2.4 ± 1.9 mEq/L for BUN <25.2 mg/dL; *P* = 0.0002; Fig. 3b).

## Discussion

In the present post hoc analysis, greater body weight and lower BUN values were identified as significant predictive factors of the pharmacological action of tolvaptan as measured by 24-h urine volume. Cutoff points of ≥500 and <500 mL were calculated to identify patients with and without an initial change in urine volume. Cutoff points of body weight of ≥59.4 and <59.4 kg (AUC, 0.66) and BUN of ≥25.2 and <25.2 mg/dL (AUC, 0.69) were predictive. Body weight can be influenced by several factors, including water intake, sex, and height. Thus, body weight alone may not be the most appropriate predictive factor. BUN, on the other hand, is a useful parameter for establishing renal function, and is affected by protein intake, gastrointestinal bleeding, and dehydration [17]. The difference between body weight of <59.4 and ≥59.4 kg in the proportion of patients with a change in initial urine volume of ≥500 mL was approximately 20 % (41 vs. 59 %; Fig. 2a), whereas the difference between BUN cutoff points was 40 % (70 vs. 30 %; Fig. 2b). The small difference based on body weight suggests that body weight may not be a factor in the pharmacological action of tolvaptan. While the difference for BUN was numerically large, the reason was not clearly evident. Therefore, we performed additional analysis to confirm the reliability of the data, and found a clear relationship among the variables.

Tolvaptan has been approved worldwide in patients with hyponatremia. In this study, we evaluated changes in serum sodium level based on the cutoff values for body weight and BUN value. The results revealed no significant difference in the effect of tolvaptan based on body weight of <59.4 and ≥59.4 kg, although an increase in serum sodium was observed in both (Fig. 3b), indicating that tolvaptan was effective regardless of body weight. On the other hand, a significant increase in serum sodium was observed in patients with BUN values of <25.2 vs. ≥25.2 mg/dL at baseline. However, because the clinical trials in our analysis were phase 2 and 3 trials, patients with severe liver cirrhosis were not included, and therefore the cutoff points, including BUN, were relatively moderate values. Investigations of patients in real-world settings are needed in the future to identify reliable cutoff points. In addition, tolvaptan use was not listed as a predictive factor. In a previous report, tolvaptan use was found to be the most reliable predictive factor of pharmacological action, although age and BUN were selected as minor factors [18]. Based on our research, a more powerful predictive factor than tolvaptan use has not been found to date.

Our analysis showed that an increase in initial urine volume of ≥500 mL with tolvaptan led to an improvement in ascites-related clinical symptoms on day 7, despite insufficient response to conventional diuretics including furosemide and spironolactone. Moreover, resolution of ascites-related clinical symptoms was observed in some patients with an increase in initial urine volume of ≥500 mL, while no resolution of symptoms was found among patients with an initial urine volume increase of <500 mL (Table 4). An improved general state—a meaningful evaluation point for confirming a change in patients’ impression of the effects of tolvaptan—was also reported at a high rate, suggesting that patients suffering troublesome symptoms might benefit from long-term tolvaptan use. Increased urine volume has been reported to be associated with a reduction in ascites volume, which may resolve some troublesome ascites-related clinical symptoms [12, 19, 20]. Bloating, loss of appetite, malaise, sensation of pressure when in a decubitus position, and difficulty breathing are ascites-related clinical symptoms commonly associated with liver cirrhosis. Patients enrolled in this cohort received tolvaptan administration for 7 days, and thus we cannot evaluate long-term outcomes including survival and prognosis. However, a clinically meaningful outcome was achieved even with short-term administration of tolvaptan. A reduction in body weight and increase in urine volume are not clinical benefits, but they are parameters for evaluating the efficacy of diuretics. The results of this post hoc analysis show that short-term administration of tolvaptan led to an improvement in ascites-related symptoms. Furthermore, the higher increase in initial urine volume was of clinical benefit as a useful parameter for predicting improvement or resolution of troublesome symptoms .

A limitation of this study was that our analysis included only those patients with mild liver cirrhosis. Because the clinical trials were conducted to approve a new indication in Japan, patients with severe liver cirrhosis were excluded. A post-market surveillance has now been conducted, and analysis using a real-world population is possible. In the future, results of a similar analysis will be provided.

In conclusion, we found that changes in urine volume were affected by both baseline body weight and BUN in tolvaptan-treated patients. Higher urine output was associated with improvement in ascites-related clinical symptoms.
